# Efficacy, chondrotoxicity and plasma concentrations of tramadol following intra-articular administration in horses undergoing arthroscopy: preliminary findings

**DOI:** 10.1080/01652176.2018.1546963

**Published:** 2019-02-18

**Authors:** Alessandra Di Salvo, Elisabetta Chiaradia, Giorgia della Rocca, Mario Giorgi, Francesco Mancini, Maria Luisa Marenzoni, Maria Beatrice Conti, Sara Nannarone

**Affiliations:** a Department of Veterinary Medicine, University of Perugia, Perugia, Italy;; b CSCS-Centro di Studi del Cavallo Sportivo, University of Perugia, Perugia, Italy;; c CeSDA-Centro di Studio sul Dolore Animale, University of Perugia, Perugia, Italy;; d Department of Veterinary Sciences, University of Pisa, Pisa, Italy

**Keywords:** Horse, equine, tramadol, chondrotoxicity, efficacy, intra-articular

## Abstract

Intra-articular administration of analgesics is performed to ensure good perioperative pain management avoiding undesirable systemic effects. To evaluate the effect of intra-articular injection of tramadol on postoperative pain after arthroscopy in horses and to determine whether tramadol had a local effect. Before the in vivo study, an in vitro test was performed aiming to evaluate the viability of equine chondrocytes after exposure to various concentrations of tramadol. The concentration identified as most appropriate was used to treat the horses’ joints. Twelve horses affected by osteochondrosis were randomly assigned to two groups that were treated intra-articularly at the end of surgery with tramadol (4 mg/mL) and saline, respectively. At predetermined time-points a Composite Pain Scale was applied and blood samples were collected in order to define the extent of tramadol absorption into the systemic circulation. The Mann-Whitney test was used for statistical analysis. Serum of four out of six treated horses revealed traces of tramadol (range 10.6–19.3 ng/mL) sporadically between 0.5 and 4 hours post-treatment, while in the other two horses, no trace of drug was found. Findings suggested that any eventual effect was probably due to local action rather than systemic absorption. The pain scores obtained in tramadol-treated horses were lower between 1 and 6 hours post-administration, than those obtained in the control group, but the differences were not statistically significant. These preliminary results suggest that tramadol, at this concentration, is only mildly beneficial in the pain management of horses after arthroscopy.

## 1. Introduction

Beside chronic or degenerative conditions which may be acquired during the athletic career of horses, osteochondrosis is a common disease of young equine. It affects the growing cartilage and without a surgical intervention may lead to poor performance with a high impact on the horse industry (van Weeren and Jeffcott 2013; Naccache et al. 2018). Arthroscopy is a minimal invasive surgical technique that has become over the years a widespread practice in equine surgery because of its great advantages (reduced hospitalisation, minor post-surgical complications, etc.) compared to traditional surgery (McIlwraith 1984).

In human arthroscopic procedures, drugs (opioids, α_2_-agonists and local anaesthetics) are often administered intra-articular (IA) to relieve perioperative pain while avoiding potential undesirable effects related to systemic treatment (Joshi et al. 2000; Al-Metwalli et al. 2008; Kazak Bengisun et al. 2010). The IA administration of different classes of drugs has recently become commonplace in the care of horses (Santos et al. 2009; van Weeren and de Grauw 2010; Di Salvo et al. 2014).

The IA administration of opioids in humans is effective as opioid-receptors are present on peripheral afferent nervous fibres and their expression is up-regulated during the inflammatory process (Stein et al. 2009). The presence of opioid-receptors in the synovial tissue of horses has also been demonstrated, supporting the IA use of these drugs to manage pain in animals undergoing arthroscopic surgery (Sheehy et al. 2001).

Tramadol is a weak inhibitor of μ-opioid receptors and an inhibitor of serotonin and adrenalin reuptake (Grond and Sablotzki, 2004). Its local anaesthetic property was also demonstrated (Altunkaya et al. 2003, 2004). In human medicine tramadol is considered a valid analgesic for acute and chronic pain (Savoia et al. 2000) and in the last decade several human studies have reported good pain management following its IA administration (Akinci et al. 2005; Jazayeri et al. 2012; Faisal et al. 2013). In the study by Alagol et al. (2004), IA administration of 100 mg of tramadol resulted in longer analgesia and in minor analgesic consumption and adverse effects than when the same dose was administered IV. The authors hypothesised that analgesia was promoted by local action of tramadol, although no investigation was made to exclude the possibility that the effect was due to systemic absorption. Indeed, the lack of a high peak concentration, due to the slow absorption of tramadol from the injection site could have prolonged the analgesic effect and reduced adverse effects.

The purpose of this study was to evaluate the efficacy of IA administration of tramadol on postoperative pain after arthroscopy in horses, and to assess whether the analgesic effect could be due to a local action or to a central effect following absorption of the drug into the circulation. In order to choose the most appropriate concentration of tramadol to inject into the equine joint, the clinical study was preceded by an in vitro test to evaluate the viability of equine chondrocytes at various tramadol concentrations. In fact, several experimental studies have revealed a potential chondrotoxic effect of many drugs (local anaesthetics, α_2_-agonists and steroids; Park et al. 2011; Wernecke et al. 2015; Mancini et al. 2017), and with regards to tramadol, a recent in vitro study has evidenced negative effects on rat chondrocyte growth (Beyzadeoğlu et al. 2012).

## 2. Materials and methods

### 2.1. Equine chondrocyte primary cell cultures

Chondrocytes were isolated from healthy articular cartilage of the metacarpo/metatarso-phalangeal joints of horses obtained from a local slaughterhouse within 1–2 h of slaughter and aseptically dissected as previously reported (Mancini et al. 2017).

### 2.2. In vitro evaluation of chondrocyte viability

Cell viability was evaluated by the 3-(4,5-dimethylthiazol-2-yl)-2,5-diphenyltetrazolium bromide (MTT) assay. Cells were seeded at a density of 15 × 10^3^ cells/well in 96-well plates, allowed to adhere for 24 h at 37 °C in a humidified atmosphere of 5% CO_2_ and then exposed for 15 min to various concentrations of tramadol from 0.1 mg/mL, the concentration associated with slight histopathological changes in rat cartilage (Fatahian Dehkordi et al. 2014), to 50 mg/mL (corresponding to the product concentration available for purchase) including 0.5, 1, 3, 4, 5, 10, 15 and 25 mg/mL.

Subsequently, the drug solution was carefully aspirated, discarded and replaced with 5 mg/mL of MTT solution (Sigma-Aldrich) diluted in culture medium. The plates were incubated for 3 h at 37 °C. Then, 200 μL DMSO was added to each well. The optical density was measured at 570 nm with a correction of absorbance read at 620 nm using a Multiskan™ GO Microplate Spectrophotometer (Thermo Fisher Scientific Inc.). The cell viability was expressed as the percentage, assuming that the absorbance of control cells was 100%.

DPBS was used to dilute tramadol and as control. To verify that 15 min of DPBS exposure had no effect, the cell viability of chondrocytes maintained for the same time in culture medium was also assayed.

### 2.3. Animals and treatments

The clinical study was conducted in accordance with the Directive 2010/63/EU of the European Parliament on the protection of animals used for scientific purposes and with the approval of the Bioethical Committee of the University of Perugia (protocol number: 2015-003).

Twelve horses affected by osteochondrosis and referred to the Veterinary Teaching Hospital of the University of Perugia for arthroscopy were included in the study after obtaining owners’ written informed consent.

Age, weight, gender and joint involved are reported in Table 1. All animals were classified as ASA I or II; the radiological severity of the lesion was scored by a single radiologist as 0 = mild (lesion <1.5 cm), 1 = intermediate (lesion 1.5–3 cm) and 2 = severe (lesion >3 cm) (Table 1).

**Table 1.** Age, gender, weight, involved joint, type of lesion, scores assigned to lesion severity and surgical invasiveness of each affected joint, anaesthesia and surgery duration and time to standing in tramadol (Group T) and saline (Group S) groups.

Group	Horse	Age (years)	Sex	Weight (kg)	Joint	Type of lesion	Radiological lesion severity	Surgical invasiveness	Duration of anaesthesia (min)	Duration of surgery (min)	mL administered	Time to standing*
Group T	1	5	M	470	L&R Carpus^b^	OF	2	4	140	65	10	20
	2	3	F	430	LF fetlock	OCD	1	2	80	15	10	17
	3	1	M	280	L hock	OCD	2	1	80	14	20	36
	4	6	M	460	L hock	AF	1	3	99	40	20	21
	5	1	M	370	R hock	OCD	2	3	90	30	20	19
	6	2	F	430	L carpus	OF	2	3	115	60	10	27
Group S	1	1	M	320	L&R hocks^a^	OCD	2	2	95	45	20	30
	2	3	G	500	LF fetlock	OCD + arthropathy	2	2	150	95	10	18
	3	2	F	340	LF fetlock	Subchondral cyst	1	1	135	80	10	20
	4	10	G	580	L hock	AF	2	3	125	90	20	39
	5	3	M	600	L&R hocks^a^	OCD	2	4	167	90	20	24
	6	9	M	460	L hock	Desmopathy	0	0	150	85	20	62

* From the end of anaesthesia.

a Horse undergoing bilateral surgery; the joint with the higher score was reported, and this joint was considered for the orthopaedic evaluations.

b Only the left joint underwent to surgery and tramadol treatment.

R (right), L (left), LF (left front), OCD osteochondrosis, OF osteochondral fragmentation, AF avulsion fracture.

Horses were premedicated IV with romifidine (0.05 mg/kg BW) (Sedivet, Boehringer Ingelheim, Firenze, Italy) and methadone (0.1 mg/kg BW) (Semfortan, Dechra, Bladel, the Netherlands), induced with diazepam (0.04 mg/kg BW, IV) (Ziapam, Dechra, Torino, Italy) and ketamine (2.5 mg/kg BW, IV) (Ketavet 100, MSD Animal Health, Aprilia (LT), Italy), and maintained with isoflurane (Vetflurane,Virbac,Carros, France) in 100% oxygen. Ten minutes before the beginning of surgery, the joint was injected with 2% mepivacaine (Carbosen 20 mg/mL, Galenica Senese, Monteroni d’Arbia (SI), Italy), 20 mL if the carpus or fetlock and 30 mL if the hock was involved, providing both distension and pre-emptive local analgesia. As pre-surgical treatment, all horses received broad spectrum antibiotics (6.6 mg/kg BW of gentamycin, IV, SID; 20,000 IU/kg BW of procain penicillin IM, BID) and phenylbutazone (2.2 mg/kg BW, IV) (Fenilbutazone, Ati, Ozzano dell’Emilia (BO), Italy). The antibiotic therapy was continued for other 4 days while the anti-inflammatory drug was repeated every 24 hours for 3 days.

At the end of surgery (after skin wound closure), the horses were randomly assigned to two groups (six horses/group) that received a different IA treatment: Group T was treated with tramadol (Tramadolo Hexal AG, Hexal, Holzkirchen, Germany) at the concentration of 4 mg/mL (chosen as the most appropriate concentration after the in vitro study), Group S received 0.9% saline solution. The volume administered in both groups was 10 mL if surgery was performed on the carpus or fetlock, and 20 mL if performed on the hock.

Surgical invasiveness, scored according to ICRS Clinical Cartilage Injury Evaluation system-2000 (ICRS 2000), the duration of anaesthesia, surgery and time to standing are reported in Table 1.

### 2.4. Evaluations of treatment efficacy and of tramadol absorption in the systemic circulation

The quality of recovery from anaesthesia was assessed using a 100-point scale according to Clark-Price et al. (2008). Orthopaedic pain was evaluated at predetermined time-points (0.25, 0.5, 1, 2, 6 and 24 hours after standing) using a Composite Pain Scale (CPS) specific for orthopaedic pain in horses (Bussières et al. 2008). The original scale was modified to include additional parameters able to detect clinical signs of tramadol systemic absorption (sedation and ataxia) and other behavioural signs possibly related to pain (position in the box, head and ear position, temperament and behaviour described as alert or apathetic), as well as orthopaedic evaluations (‘tumor’, ‘calor’, lameness at walk and non-weight bearing). The total score of the modified CPS was 56 (Table 2).

**Table 2.** Parameters added to the Composite Pain Scale (CPS) of Bussières et al. (2008).

Temperament	Calm	0
	Agitated	1
	Intractable	2
Behaviour	Alert	0
	Apathetic	1
Sedation	No	0
	Yes	1
Ataxia	No	0
	Yes	1
Position in the box*	In front of the door, watching the environment	0
	Standing in the middle, watching the door	1
	Standing in the middle, watching the walls	2
	Standing in the middle, watching the back of the box	3
Head position*	Above the withers	0
	At the withers	1
	Below the withers	2
Ear position	Normally forward, frequent movements	0
	Slightly back, little movements	1
Tumor	No	0
	Mild	1
	Severe	2
Calor	No	0
	Yes	1
Lameness at walk	No	0
	Yes	1
Non-weight bearing	No	0
	Mild	1
	Severe	2
Total score		17

The possible total score of the modified CPS is 56: 39 points from the original CPS of Bussières et al. (2008) plus 17 points from the additional parameters reported above.

* According Lindegaard et al. 2010.

A single observer, blinded to the IA treatment, performed the evaluations for the entire duration of the study.

Blood samples at predefined time-points (immediately prior to tramadol administration and at 15, 30, 45, 60, 90, 120, 180, 240 and 300 minutes after drug injection) were taken to evaluate, in Group T, the concentration of tramadol and its active metabolite (M1) in the systemic circulation vs time. The analytical determination of tramadol and M1 in samples was performed using a method validated in equine plasma previously published by Giorgi et al. (2007). The lower limit of quantification of the analytical method (LLOQ) was 10 ng/mL for both analytes; the intraday coefficient of variation values (CV%) were always lower than 3.9 and 11.42% for tramadol and M1, respectively; the intraday accuracy percentages were between – 1.01 and 14.81% of nominal value for tramadol and between – 4.07 and 15.08% for M1 (this last percentage is referred to a nominal value of LLOQ).

The analytical method was able to determine the three main metabolites of tramadol (M1, N-desmethyltramadol (M2), and N,O-didesmethyltramadol (M5)) separately, but only the active metabolite (M1) was considered in this study as this is responsible for analgesic efficacy.

### 2.5. Statistical analyses

Data of cell viability were obtained from ≥4 independent experiments performed in triplicate. Data, expressed as mean of percentage ± standard deviation (SD), were analysed by one-way analysis of variance (ANOVA) followed by the Bonferroni post-hoc test.

The homogeneity of the two groups with regard to age, gender, body weight, lesion severity, surgical invasiveness, duration of surgery, anaesthesia and time required to achieve the standing position was evaluated using the Mann-Whitney test. The Shapiro-Wilk test was performed to assess whether the data obtained from the horses were normally distributed with regard to the CPS values. The Mann-Whitney test was used to find possible differences in the recovery quality and the effect of IA treatments on post-operative pain between the two groups. A statistical significance was considered for *P* values <0.05.

## 3. Results

### 3.1. Effects of tramadol on chondrocyte cell viability

**Figure 1:**
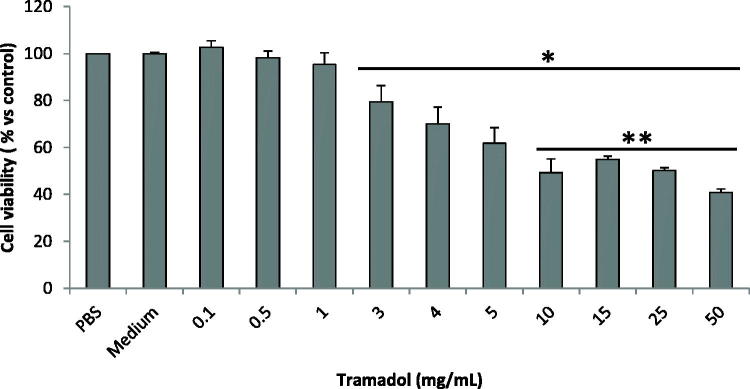
Chondrocyte viability (% vs control) exposed to different concentrations of tramadol for 15 min. Bars represent the standard deviations. *p < 0.0001 vs control (cells treated with PBS); **p < 0.05 vs concentrations of 3 and 4 mg/mL

Figure 1 shows the cell viability percentage after treatment with various concentrations of tramadol (0.1–50 mg/mL) evaluated by the MTT assay.

Compared to cells treated with PBS, tramadol induced a significant reduction of chondrocyte cell viability in a dose-dependent manner from the concentration of 3 mg/mL (*P* < 0.0001) on. The most pronounced cytotoxic effect was observed at 50 mg/mL of tramadol (40.8 ± 1.5% of viable cells). No effect was observed when the cells were exposed to 0.1–1 mg/mL, while concentrations ranging between 10 and 25 mg/mL elicited a similar effect on chondrocyte viability as 50 mg/mL (mean range of viable cells between 49.3 and 54.9%). The cell viability at concentrations between 10 and 50 mg/mL tramadol was significantly reduced (all P values were lower than 0.05) with respect to that observed at 3 and 4 mg/mL (79.4 ± 7.0% and 70.1 ± 7.1% of viable cells, respectively). No difference was observed in viability of chondrocytes exposed to 3 and 4 mg/mL.

No time-dependent toxic effects of tramadol were observed when treatment was prolonged for 30 min (data not shown).

### 3.2. Evaluations of treatment efficacy and of tramadol absorption in the systemic circulation

During surgery horse no 6 of Group S was also diagnosed with a desmopathy (Table 1), therefore it was excluded from the efficacy evaluations as this horse would also be experiencing pain associated with ligaments. As a consequence, for the purposes of the study, 6 horses were included in Group T and 5 in Group S. Horse no 1 in Group T underwent a bilateral arthroscopy but only the left joint presented an osteochondral fragmentation requiring surgical debridement, while the right one was free of pathology, therefore tramadol treatment was only administered to the first joint.

The two groups were homogenous according to age, gender, body weight, lesion severity, surgical invasiveness and duration of anaesthesia. A statistically significant difference was observed for surgery duration (*P* = 0.017), that was longer in Group S.

During recovery from general anaesthesia, one horse in Group S required administration of romifidine (0.01 mg/kg BW) as a sedative; therefore, it was excluded from the time and recovery score evaluation; for all other horses, no difference was detected between the groups.

**Figure 2:**
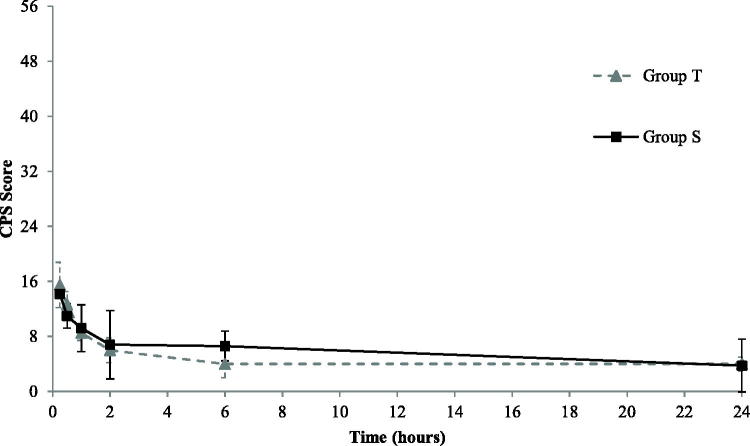
Mean ± S.D. of obtained scores vs time following application of a composite pain scale (CPS) (Bussières et al.,^2008^, modified) in Group T (grey dotted line, triangle [-▲-]) and Group S (black solid line, square [-■-]). The value of 56 on the *y*-axis represents the maximum score obtainable with the CPS and the bars represent the standard deviations. The mean ± S.D. of CPS scores are calculated on 5 subjects (S group) and 6 subjects (T group) except for the 24 h time-point in which one horse in each group was discharged before the end the study at the request of the owner.

The CPS values obtained within a group were normally distributed at all evaluation time points except for that at 30 min in group T and 60 min in Group S. The CPS scores *vs* time points for each group are represented in Figure 2. The mean ± SD of CPS at 24 h was calculated on 4 subjects (S group) and 5 subjects (T group) because one horse in each group was discharged prior to the end of the study at request of the owners. Overall, pain scores, from the first hour up to 6 h, were slightly lower in Group T than in Group S, but no statistically significant differences were observed at any time point. No rescue analgesia was deemed necessary in either group.

No traces of tramadol were found in the plasma samples of two out of six treated horses, while in the other animals only very low tramadol concentrations, ranging from 10.6 to 19.3 ng/mL, were sporadically observed from 0.5 to 4 h post-treatment. The presence of M1 was never detected.

## 4. Discussion

Intra-articular treatments are routinely used to prevent or relieve pain in humans and veterinary patients, but in vitro studies have often shown deleterious effects on chondrocyte viability (Park et al. 2011; Wernecke et al. 2015; Mancini et al. 2017). Regarding tramadol, some recent studies reported signs of inflammation in rat articular cartilage following IA injection of the drug (Fatahian Dehkordi et al. 2014; Kola et al. 2015). An in vitro study evidenced that tramadol may be more harmful to rat chondrocytes than other chondrotoxic drugs such as bupivacaine and levobupivacaine (Beyzadeoğlu et al. 2012). To choose a safe IA concentration of tramadol for the treatment of equine joint pain, chondrocyte cultures were exposed to various concentrations. The exposure of equine chondrocytes to 50 mg/mL of tramadol resulted in marked toxicity, and similar signs were observed at concentrations of 10 and 25 mg/mL. For this reason, administering the drug at these concentrations was considered inappropriate for the in vivo study. A higher percentage of cell viability, statistically different from that observed between 10 and 50 mg/mL, was observed at 3 and 4 mg/mL; therefore, a concentration of 4 mg/mL of tramadol was chosen, despite the viability of chondrocytes being significantly reduced with respect to the controls. However, it is important to emphasise that the in vitro results do not necessarily reflect the in vivo behaviour also considering tramadol. Indeed, the dilution of the drug in the synovial fluids and/or its absorption in the systemic circulation may reduce the toxic effects on chondrocytes (Webb and Ghosh, 2009). Wernecke et al. (2015) emphasised that while in vitro studies revealed chondrotoxicity following IA administration of corticosteroids, in vivo studies showed a protective effect of the same drugs on articular cartilage. The concentration of tramadol used in our study is similar to that reported as effective in humans (range 2.5–8 mg/mL through the IA route) for pain management following arthroscopy (Alagol et al. 2004; Akinci et al. 2005; Hassan and Khalil 2005), and at this time, to our knowledge, there is no evidence of deleterious side effects on treated joints in the literature.

As recovery is known as a crucial phase in equine anaesthesia, we hypothesised that a locally injected drug, such as tramadol, could represent a further contribution to a safe recovery. Recovery should be smooth, coordinated and devoid of anxiety and incoordination, that may arise from pain at the operated site (Santos et al. 2003; Clark-Price, 2013). In our study we did not observe any significant difference in terms of quality and time of recovery between groups, but the small sample size as well as the use of a multimodal pre-emptive analgesic protocol in both groups may account for this lack of evidence.

To assess the presence of pain in the post-operative period we used the CPS described by Bussières et al. (2008) for orthopaedic pain in horses modified by adding some behavioural and orthopaedic indicators. Horse behaviour, such as position in the box, interactive behaviour and head and ear position, although not specific for orthopaedic pain, is considered indicative of equine pain (Dalla Costa et al. 2014; Gleerup et al. 2015; Gleerup and Lindegaard, 2016), therefore they were combined with more specific orthopaedic findings such as ‘tumor’, ‘calor’ and lameness at walk that are typical signs of inflammation which can cause pain. Moreover, other parameters, such as sedation and ataxia, were included in our CPS with the intent to verify the presence of clinical signs related to a systemic action of tramadol. In a previous study, Di Salvo et al. (2014) observed sedation and a significant reduction of respiratory rate following IA administration of xylazine in horses after arthroscopy compared to control group, indicating a presumed systemic effect.

No significant differences in the CPS values were observed in the two groups, although the pain scores obtained from the first hour following standing were slightly lower in Group T than in Group S, and this difference persisted up to 6 h. This result, together with the lack of a requirement for rescue analgesia in both groups, might indicate that the perioperative analgesic protocol was able to fully manage pain in the post-surgical period. As a consequence, only a limited further benefit could have been observed in the tramadol-treated group. However, the lack of a statistically significant difference between the two groups could also be due to a type II error, namely, that difference between the two treatments exists, but it is not evident due to the low number of animals enrolled in the study (Hofmeister et al. 2007).

In a study conducted by Jahromi et al. (2016), a statistically significant difference in pain scores of horses treated with IA tramadol (2 mg/kg BW) compared with horses treated with saline was observed at each time point. Nevertheless, the different pre-anaesthetic protocol adopted by Jahromi et al. (2016), without α_2_-agonists, opioids and NSAIDs, may have been responsible for a lower analgesia in the post-operative period, allowing the difference to be detected between the two groups. Furthermore, a considerably higher dose of tramadol was injected compared to that used in our study (2 mg/kg BW vs a range of 0.09–0.29 mg/kg BW by virtue of the fixed concentration of tramadol at 4 mg/mL). In a preceding study, Jahromi et al. (2011) observed an increase in alkaline phosphatase, aspartate amino transferase and lactic dehydrogenase activity in the equine synovial fluid after IA administration of 2 mg/kg BW tramadol, compared with those obtained before the IA injection. These values were similar or even higher than those obtained following the IA injection of 2% lidocaine (Jahromi et al. 2011). The increase in these parameters in equine synovial fluid was correlated with the presence of articular inflammation and necrotic tissue (Bashandy et al. 2014). Although IA lidocaine administration is a widespread practice to provide analgesia in human and in veterinary medicine (Arai et al. 2005; Van Vynckt et al. 2010; Di Salvo et al. 2015), and a single IA administration in the equine joint seems to be safe in terms of adverse effects (Piat et al. 2012), several in vitro studies (Jacobs et al. 2011; Miyazaki et al. 2011; Di Salvo et al. 2016) showed chondrotoxic activity of this drug, thus prompting the authors to warn about its use through the IA route. Similarly, according to our results on tramadol chondrotoxicity, its use at high concentrations should be performed with caution. If a dose of 2 mg/kg BW of tramadol had been used in our study (this treatment would have only been possible in the joints injected with 20 mL), the administered concentrations would have been near or equal to 50 mg/mL, which induces a cell mortality of about 60%, although, as mentioned previously, what happens in vitro in terms of chondrotoxicity is not necessarily superimposable to what occurs in vivo.

The plasma concentrations of tramadol and its metabolite able to produce a systemic analgesic effect have not yet been established in the horse. If these concentrations were equal to those reported in humans (287.7 ng/mL, median value, for tramadol and 84 ± 34 ng/mL, mean ± SD, for M1), the concentrations observed in our study would have been a long way off producing a systemic effect (Lehmann et al. 1990; Grond et al. 1999). If future studies, enrolling a greater number of subjects/group, will confirm the efficacy of IA tramadol at 4 mg/mL, it will be possible to speculate that the drug’s efficacy is due to its local action. In fact, the absence of detectable tramadol concentrations in plasma in two horses and the very low amounts found in the other four subjects allow us to exclude a systemic effect.

## 5. Conclusions

These preliminary data on the efficacy of IA tramadol at 4 mg/mL in horses undergoing arthroscopy suggest that this concentration may be inadequate to enhance post-operative pain management, but further studies with a larger number of subjects are warranted.
